# Nivolumab-Induced Organizing Pneumonia in Management of Non-small Cell Lung Carcinoma: A Case Report

**DOI:** 10.7759/cureus.39217

**Published:** 2023-05-19

**Authors:** Tehmina Habib, Mohammad Abu-Abaa, Diana Kolman-Taddeo

**Affiliations:** 1 Internal Medicine, Capital Health Regional Medical Center, Trenton, USA

**Keywords:** organizing pneumonia, pneumonitis, ipilimumab, nivolumab, non- small cell carcinoma

## Abstract

Nivolumab is an immune checkpoint inhibitor (ICI) that has proven efficacy in managing certain malignancies, including non-small lung carcinoma. In this case, we present a 53-year-old female patient diagnosed with metastatic non-small lung carcinoma. After management with radiation (both external beam and brachytherapy) and tumor debulking by bronchoscopic cryotherapy, she developed an initial pneumonitis attributed to nivolumab and ipilimumab. This was successfully managed with steroid therapy and allowed nivolumab monotherapy to restart. However, several months later, she developed organizing pneumonia, prompting immunotherapy discontinuation and initiation of corticosteroid therapy. This case serves as a reminder to clinicians that although ICIs constitute a novel, effective therapy for certain malignancies, immunological side effects can be debilitating and prevent continued immunotherapy. Through this case, we aim to review the literature about this rare side effect of nivolumab-induced pneumonitis, risk factors, diagnosis, and management.

## Introduction

Several immune checkpoint inhibitors (ICIs) have been approved for various tumors. These include cytotoxic T lymphocytes associated protein 4 (CTLA-4) inhibitors, e.g. ipilimumab, programmed cell death protein type 1 (PD-1) inhibitor, e.g. nivolumab and pembrolizumab, programmed cell death protein ligand type 1 (PD-L1) inhibitor e.g. atezolizumab, durvalumab and avelumab [1]. Nivolumab induces an immune response against tumors by binding to PD-1 receptors as well as its ligands and has a favorable safety profile in the management of patients with malignant melanoma, non-small cell carcinoma, Hodgkin lymphoma, renal cell carcinoma, and head and neck cancers [2,3]. As ICIs are becoming more common, their unique side effects, named “immune-related adverse events,” are becoming more common. This case aims to remind clinicians of this complication as it can be confused with underlying tumor progression [4].

## Case presentation

**Figure 1:**
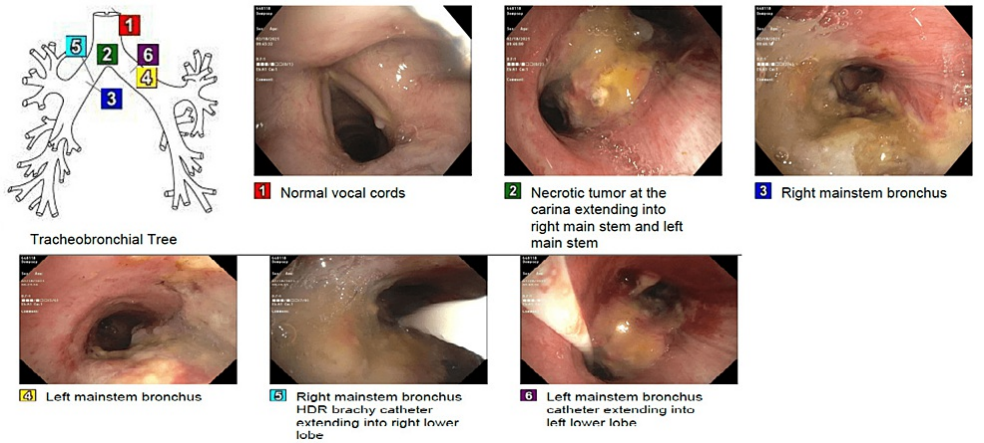
Bronchoscopy Bronchoscopic images showing endobronchial tumor (images number 2) and placement of brachytherapy catheter (image number 5).

**Figure 2:**
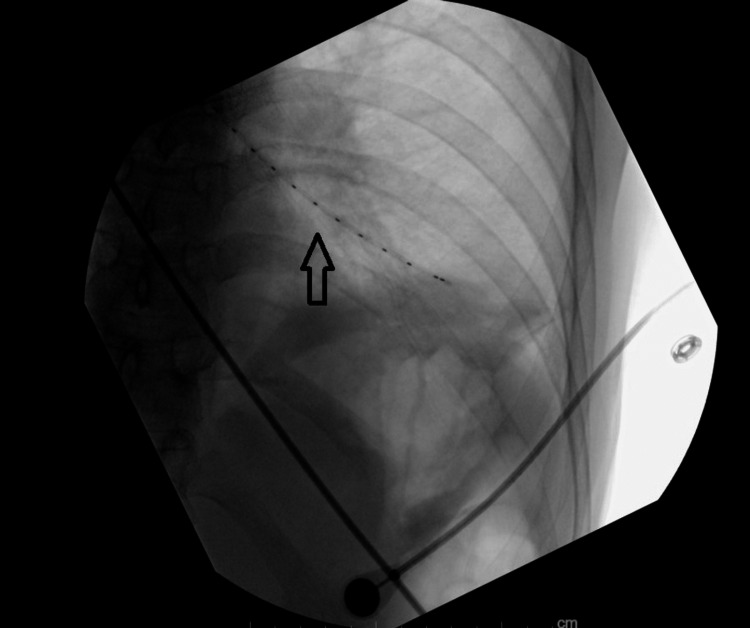
Chest X-Ray Brachytherapy catheter (arrow).

In February 2021, a 53-year-old female patient was referred by her primary care physician to the pulmonary consultation service to evaluate chronic cough, shortness of breath, and over 20 pounds of weight loss and fatigue. These have been worsening over the month before her presentation with limitations of her daily activities. Her medical history was significant for her long-term smoking history and chronic obstructive lung disease (COPD). In the office, vital signs included a temperature of 37.8 degrees Celsius, a heart rate of 108 beats per minute with a sinus rhythm, blood pressure of 140/80 mmHg, respiratory rate of 18 cycles per minute, and SpO_2_ of 99% on room air. Chest X-ray obtained by her primary care physician showed a large endobronchial lesion affecting the distal trachea and left main bronchus with mediastinal lymphadenopathy with a 1cm in diameter left lower lobe pulmonary nodule. On a physical exam, she was emaciated with decreased air entry bilaterally basally, and no evidence of lymphadenopathy was appreciated on the exam. Otherwise, her exam was unremarkable. Rigid bronchoscopy with tumor debulking was pursued, which showed 25% occlusion of the left main stem with necrotic tumor, 30% occlusion of the right main stem with necrotic tumor, and brachytherapy catheter placed into the left main stem, and also a second catheter was placed into the right main stem (Figure 1, 2).

**Figure 3:**
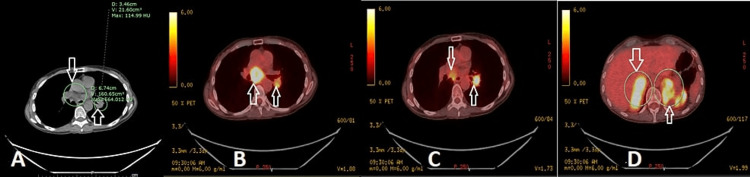
Positron emission tomography(PET)/Computed tomography (CT) Positron emission tomography (PET)/Computed tomography (CT) scan showing evidence of hypermetabolic left hilar mass (B and C) with metastasis to bilateral adrenal glands (D).

**Figure 4:**
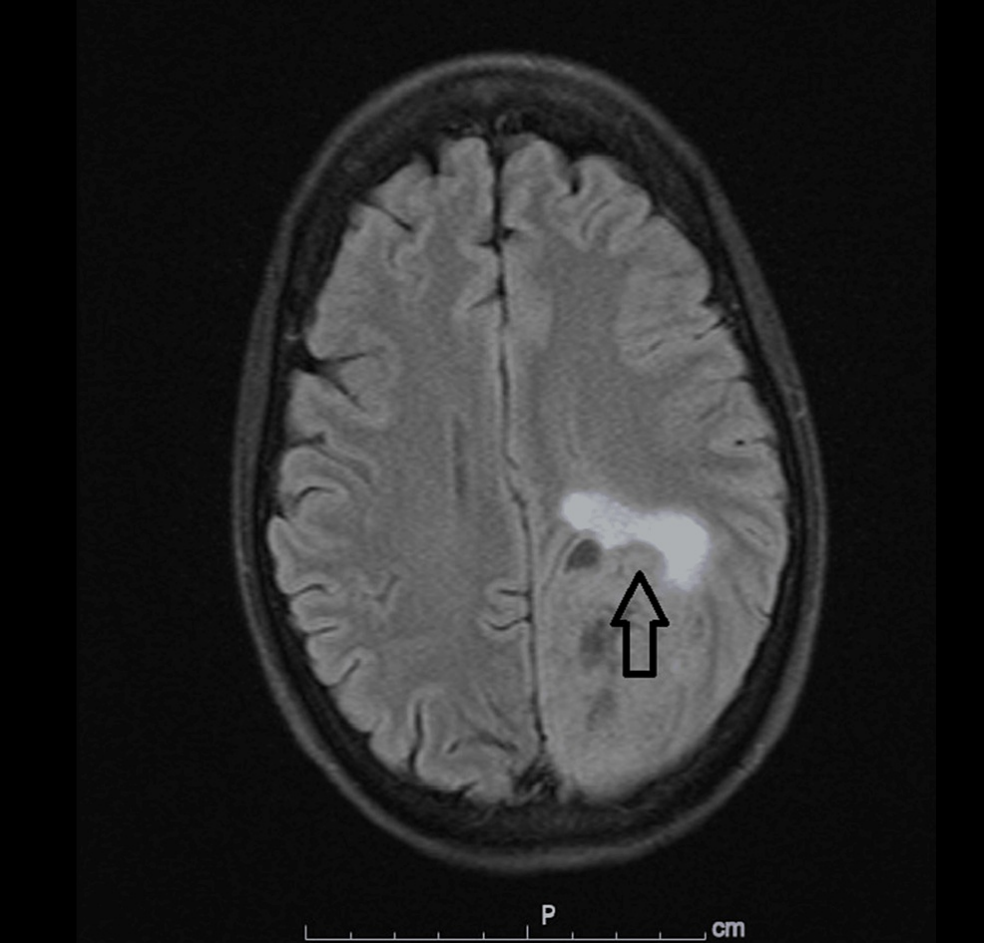
MRI Brain Magnetic resonance imaging (MRI) brain also showed evidence of a 6.4 cm left parietal-occipital mass (arrow).

**Figure 5:**
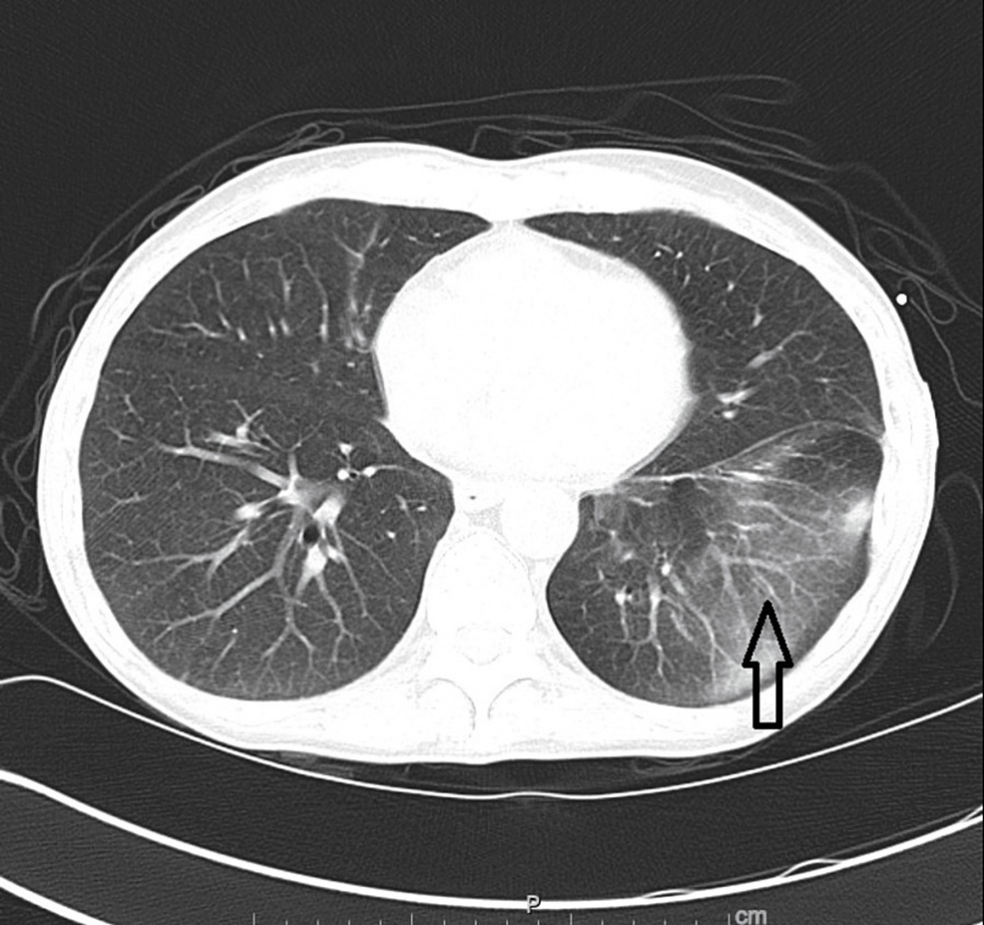
CT scan of chest. Chest CT scan showing faint ground glass opacities throughout the lung (arrow).

Positron emission tomography (PET)/Computed tomography (CT) scan showed evidence of hypermetabolic left hilar mass with metastasis to bilateral adrenal glands (Figure 3). She also received external beam radiation therapy to bilateral lungs. Pathological examination of a biopsy obtained during bronchoscopy showed poorly differentiated non-small cell carcinoma, and an adrenal biopsy also proved lung carcinoma metastases. She also developed right-sided visual loss with right-sided weakness and neglect, and Magnetic resonance imaging (MRI) brain also showed evidence of a 6.4 cm left parietal-occipital mass (Figure 4). She underwent a left-sided craniotomy, CyberKnife therapy, and tumor resection. Two months after her initial presentation (April 2021), she was also started on monthly nivolumab/Ipilimumab immunotherapy. Two months later, this was interrupted by the development of pneumonitis with a chest CT scan showing faint ground glass opacities throughout the lung that improved with prednisone therapy (Figure 5).

**Figure 6:**
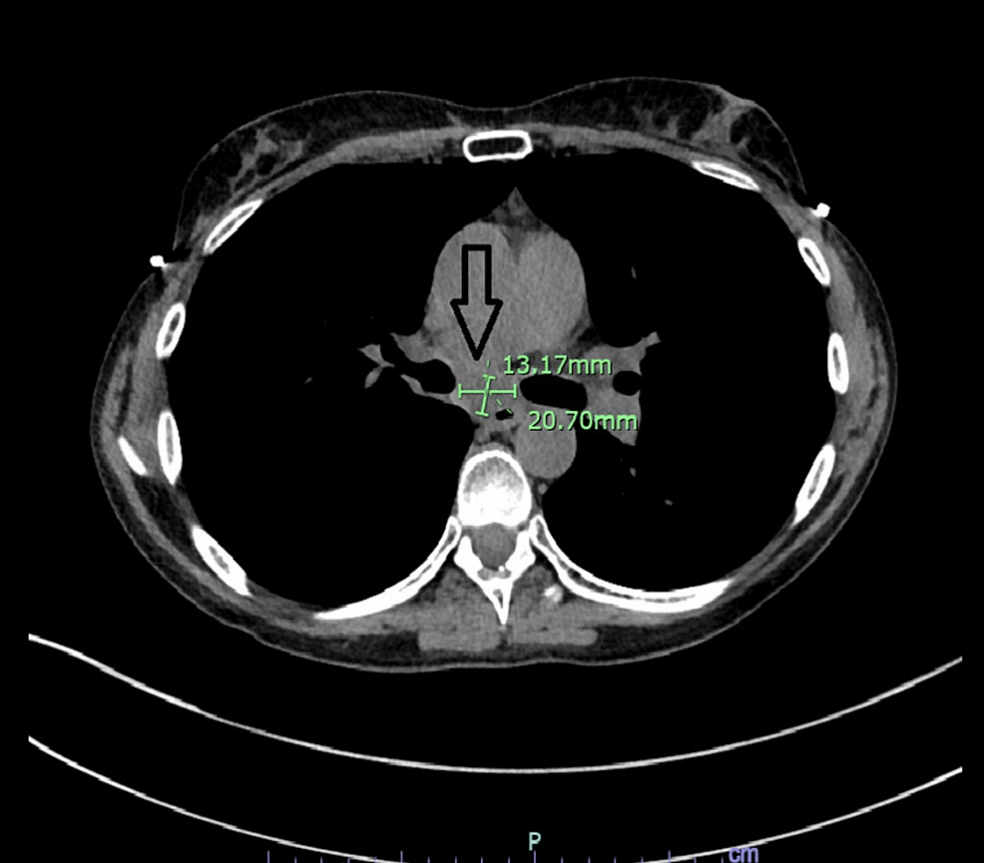
Followup CT chest A follow-up CT scan of the chest showed a slightly reduced size of the subcarinal mass (arrow).

**Figure 7:**
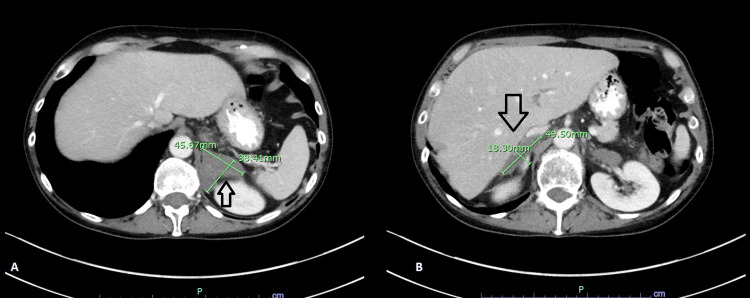
CT Abdomen A follow-up CT abdomen showed a slight reduction in the size of adrenal metastases (arrows in A and B).

**Figure 8:**
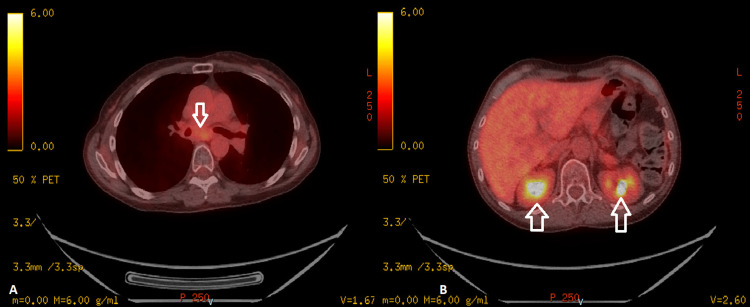
Follow-up PET Scan A follow-up PET scan showed improvement in the size of hilar mass (arrow in A) and adrenal metastases (arrow in B).

Two months later (June 2021), she resumed only on monthly nivolumab. A follow-up CT scan of the chest showed a slightly reduced size of the subcarinal mass (Figure 6). She also had other bronchoscopies aiming to debulk the endobronchial tumor with cryotherapy. A slight reduction in the size of adrenal metastases was also evident (Figure 7). Nine months after the initial presentation (November 2021), the PET scan showed improvement in the size of the hilar mass and adrenal metastases (Figure 8).

**Figure 9:**
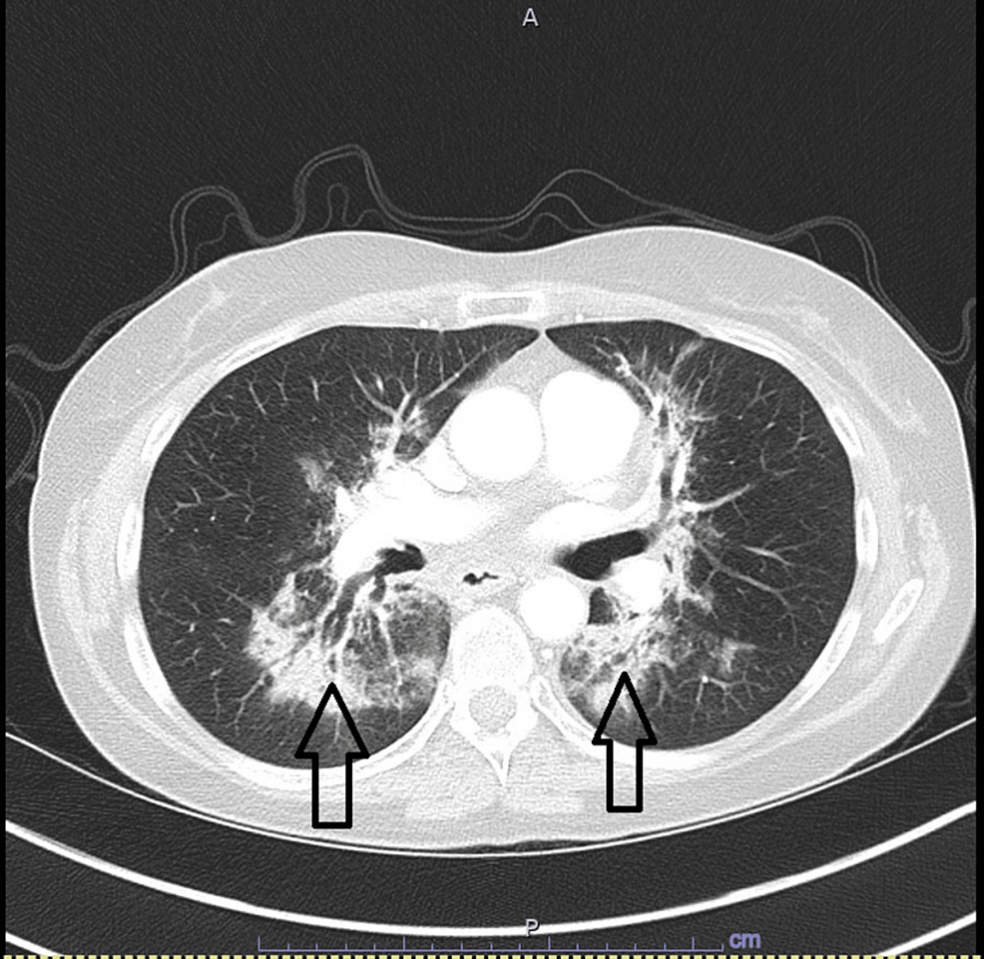
Chest CT Scan CT chest that shows the progression of diffuse bilateral ground glass opacities (arrows).

**Figure 10:**
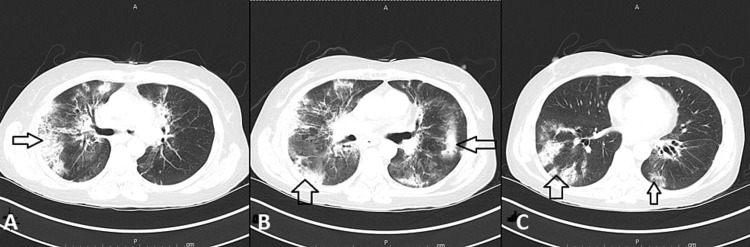
Chest Computed Tomography Even further progression was seen in a repeat CT scan of the chest in December 2022 (Arrows in A, B, and C).

**Figure 11:**
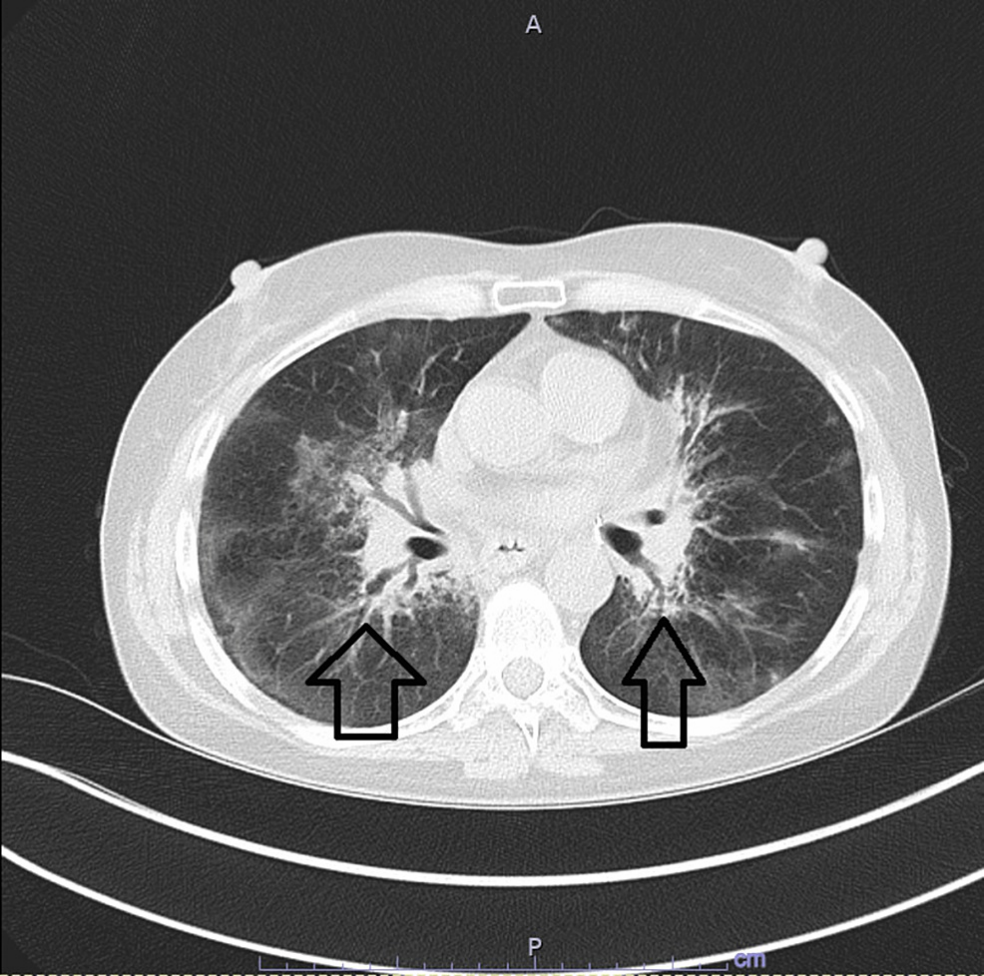
Improvement of Radiological CT Chest Findings A follow-up chest CT scan in January 2023 showed radiological improvement of infiltrative lung lesions (arrow).

Immunotherapy was complicated by new onset hypothyroidism with initially elevated TSH at 456 U/L, and she was initiated on thyroid hormone therapy. In September 2022, her respiratory symptoms of cough and shortness of breath were progressive, prompting a repeat CT chest that showed progression of diffuse bilateral ground glass opacities (Figure 9). Even further progression was seen in a repeat CT scan of the chest in December 2022 (Figure 10). A lung biopsy was obtained, whose histological examination showed evidence of chronic interstitial inflammation with fibrosis and some atypical pneumocytes with enlarged nucelli and hyperchromasia with no malignancy evidence. Immunotherapy was held, and the patient was initiated on a prednisone course. A follow-up chest CT scan in January 2023 showed radiological improvement of infiltrative lung lesions (Figure 11).

## Discussion

The incidence of immune checkpoint inhibitors (ICIs) related pneumonia is 1-5%, and ICIs-related pneumonitis is 1-7% in clinical practice [1]. The risk of immune-mediated pneumonitis related to the use of programmed cell death protein type 1 (PD-1) inhibitors such as Nivolumab is estimated at 3.02%, which is higher than that of cytotoxic T lymphocyte-associated protein type 4 (CTLA-4) inhibitors (1.06%) and less than that of a combination of PD-1 and CTLA-4 inhibitors (7.09%). On the other hand, the incidence of ICIs related pneumonia in PD-1 inhibitors is 2.23%, which is lower than that in CTLA-14 inhibitors (5.47%) [5]. Nivolumab-related pneumonitis has a mortality rate of 17.2% [6]. It is reported that Nivolumab-related pneumonitis is more prevalent among those with non-small cell carcinoma and renal cell carcinoma than those with melanoma [6]. The incidence of pulmonary complications was also higher in those receiving the combination of ipilimumab and nivolumab [7].

Immune checkpoint inhibitors (ICIs) related pneumonitis is considered secondary to chronic interstitial inflammation, similar to collagen vascular diseases [8]. This was attributed to the malfunction of regulatory T cells in the pulmonary interstitium [9]. Unlike our patients, pre-existing interstitial lung disease is considered a risk factor for nivolumab-related pneumonitis [10]. Other reported risk factors of nivolumab-induced pneumonitis include male gender and smoking history [11]. Ethnicity, especially Japanese patients, was also reported as more susceptible [12]. Prior history of chemotherapy, lung cancer, and chronic obstructive lung disease (COPD) is also reported as a risk factor [13,7].

Immune-mediated adverse events of ICIs include cardiac, neurological, and endocrine side effects, nephritis, pneumonitis, and colitis. Pneumonitis is relatively uncommon but can be life-threatening [14]. Clinical presentation is usually non-specific, and most cases present with progressive dry cough and shortness of breath [15]. Other symptoms can also include chest pain and hemoptysis [7]. Diagnosis is suggested by computed tomography (CT) scan findings of ground glass opacities and consolidations [14]. Diagnosis requires the exclusion of other etiologies, especially infectious ones, which require bronchoscopy and bronchoalveolar lavage [7]. A retrospective study found that multiple radiological patterns can be seen in nivolumab-associated pneumonitis, including cryptogenic organizing pneumonia (COP), which is the most common pattern in imaging studies seen, with a median onset of 2.6 months from the start of the ICI [10,14]. More than 63% of patients with nivolumab-related pneumonitis had radiological features typical for drug-induced pneumonitis with ground glass opacities bilaterally dominant in the lung contralateral to the tumor. Less commonly, over 36% of them had atypical features with either ground glass opacities confined to the surrounding area of the tumor “peritumoral infiltration”, abnormal opacities confined to ipsilateral lung, exacerbation of radiation fibrosis, and/or intensified infection [6].

The preferred treatment of Nivolumab-related pneumonitis is corticosteroids. Radiological patterns can predict the response to the therapy, with cryptogenic organizing pneumonia (COP) and non-specific interstitial pneumonia (NSIP) having better responses to steroids than idiopathic pulmonary fibrosis and usual interstitial pneumonia (UIP) [16].

## Conclusions

ICIs are gaining more popularity in managing certain malignancies, including lung carcinomas. However, uncommon side effects are becoming more prevalent, so clinicians should remain vigilant. Nivolumab-induced pneumonia and pneumonitis are uncommon side effects with non-specific presentation. Diagnosis requires the exclusion of other etiologies, and radiological findings are variable. Those with lung cancer, prior radiation, interstitial lung disease, COPD, chemotherapy, and smoking history have a higher risk of complications. Although there is no consensus regarding management, a corticosteroid is the most commonly used first line. Those with COP patterns are likely to respond better to steroids.

## References

[1] Su Q, Zhu EC, Wu JB, Li T, Hou YL, Wang DY, Gao ZH (2019). Risk of pneumonitis and pneumonia associated with immune checkpoint inhibitors for solid tumors: a systematic review and meta-analysis. Front Immunol.

[2] Topalian SL, Hodi FS, Brahmer JR (2012). Safety, activity, and immune correlates of anti-PD-1 antibody in cancer. N Engl J Med.

[3] Brahmer J, Reckamp KL, Baas P (2015). Nivolumab versus docetaxel in advanced squamous-cell non-small-cell lung cancer. N Engl J Med.

[4] Yin B, Xiao J, Li J, Liu X, Wang J (2020). Immune-related organizing pneumonitis in non-small cell lung cancer receiving PD-1 inhibitor treatment: a case report and literature review. J Cancer Res Ther.

[5] Robert C, Schachter J, Long GV (2015). Pembrolizumab versus ipilimumab in advanced melanoma. N Engl J Med.

[6] Baba T, Sakai F, Kato T (2019). Radiologic features of pneumonitis associated with nivolumab in non-small-cell lung cancer and malignant melanoma. Future Oncol.

[7] Kalisz KR, Ramaiya NH, Laukamp KR, Gupta A (2019). Immune checkpoint inhibitor therapy-related pneumonitis: patterns and management. Radiographics.

[8] Chow LQ (2013). Exploring novel immune-related toxicities and endpoints with immune-checkpoint inhibitors in non-small cell lung cancer. Am Soc Clin Oncol Educ Book.

[9] Pardoll DM (2012). The blockade of immune checkpoints in cancer immunotherapy. Nat Rev Cancer.

[10] Koyama N, Iwase O, Nakashima E (2018). High incidence and early onset of nivolumab-induced pneumonitis: four case reports and literature review. BMC Pulm Med.

[11] Kato T, Masuda N, Nakanishi Y (2017). Nivolumab-induced interstitial lung disease analysis of two phase II studies patients with recurrent or advanced non-small-cell lung cancer. Lung Cancer.

[12] Kudoh S, Kato H, Nishiwaki Y (2008). Interstitial lung disease in Japanese patients with lung cancer: a cohort and nested case-control study. Am J Respir Crit Care Med.

[13] Nishino M, Ramaiya NH, Awad MM (2016). PD-1 inhibitor-related pneumonitis in advanced cancer patients: radiographic patterns and clinical course. Clin Cancer Res.

[14] Nishino M, Sholl LM, Hodi FS, Hatabu H, Ramaiya NH (2015). Anti-PD-1-Related Pneumonitis during cancer immunotherapy. N Engl J Med.

[15] Nishino M, Giobbie-Hurder A, Hatabu H, Ramaiya NH, Hodi FS (2016). Incidence of programmed cell death 1 inhibitor-related pneumonitis in patients with advanced cancer: a systematic review and meta-analysis. JAMA Oncol.

[16] Belloli EA, Beckford R, Hadley R, Flaherty KR (2016). Idiopathic non-specific interstitial pneumonia. Respirology.

